# Enhancing rate of water absorption in seeds via a miniature surface acoustic wave device

**DOI:** 10.1098/rsos.181560

**Published:** 2019-03-13

**Authors:** Kiing S. Wong, Lillian Lee, Leslie Y. Yeo, Ming K. Tan

**Affiliations:** 1 School of Engineering, Monash University Malaysia, 47500 Bandar Sunway, Selangor, Malaysia; 2 Micro/Nanophysics Research Laboratory, RMIT University, Melbourne, Victoria 3001, Australia

**Keywords:** microfluidics, surface acoustic waves, acoustic streaming, germination, water absorption

## Abstract

Seeds, which are high in protein and essential nutrients, must go through a hydration process before consumption. The ability to rapidly increase water absorption can significantly reduce the soaking time as well as the amount of energy needed for cooking seeds. Many studies in the literature employ high-power (10^2^ W) low-frequency (10^4^ Hz) ultrasound; although their results are very promising where more than 100% increase in water content can be obtained between the treated and untreated seeds, the high-power and low-frequency ultrasound often causes acoustic cavitation under high intensity, which can severely disrupt the cell walls and damage the seeds. In our study, however, we demonstrate that treating the seeds via a miniature surface acoustic wave device, which operates at low-power (10^0^ W) and high-frequency (10^7^ Hz) range, gives rise to a higher water absorption rate without the acoustic cavitations. By comparing the water content between the treated and untreated seeds, an increase of up to 2600% (for chickpeas) and 6350% (for mung bean) can be obtained after 60 min. A significantly higher water absorption in mung beans can be attributed to the larger pore size when compared with the acoustic wavelength in water, enabling an efficient transmission of acoustic wave inside the pores. Our results also indicate that the germination time can be reduced by half for treated seeds as compared to the untreated seeds.

## 1. Introduction

Seeds are an important protein source and are one of the staple foods in many countries. Prior to cooking, dry seeds often require an overnight soaking at room temperature to increase their water content. Another effective way to increase the water absorption rate is to raise the temperature of the water. For instance, the water content can increase significantly during slow cooking within a few hours. However, this process is often energy inefficient due to the heat dissipation to the surroundings. Therefore, the ability to rapidly increase the water content in seeds is useful.

Most reported studies have been focused on seed germination. Nevertheless, since water absorption is the first step in germination, increasing the rate of water absorption increases the rate of germination. One of the effective techniques is to use ultrasound to enhance the water absorption in seeds or grains [1–8]. A recent study suggested the enhancement of water absorption in seeds after treatment with ultrasound can be attributed to two main mechanisms [9]: formation of micro-cavities due to acoustic cavitation, and increase in diffusion rate due to the flow induced by the acoustic wave in the liquid as well as the vibration of the seeds. Goussous *et al.* [1] reported an increase in the water content for chickpea (≈25%), wheat (≈40%), pepper (≈110%) and watermelon (≈30%) seeds after treating the seeds to 40 kHz and 100 W ultrasounds in less than 15 min, as compared to seeds without ultrasound treatment. After prolonged treatment (60 min), for chickpea and watermelon seeds, they found that there was no significant difference in the water content between the treated and untreated seeds. Interestingly, for wheat and pepper seeds, the water content for untreated seeds was higher than the treated seeds. As the acoustic power was relatively high, after prolonged treatment, the pepper seed cells were lysed due to the strong acoustic cavitation that led to the disruption of cell wall. On the other hand, the effect on chickpea and watermelon seeds was not significant, suggesting these seeds were able to remain intact after prolonged treatment under high power ultrasound. A similar finding was reported by Yildirim *et al.* [5], for chickpea under 60 min treatment with 40 kHz and 100 W ultrasound. The increase in the water content was less than 3% between the treated and untreated chickpeas at 30°C. Other studies which also demonstrated the use of high-power (in the order of 10^2^ W) low-range frequency (of the order of 10^4^ Hz) ultrasound to obtain higher water content for seeds include: Miano *et al.* [4,8] reported up to 20%, and 50%, increase in water content after treated corn kernels, and mung beans, to a 41 W L^−1^, 25 kHz ultrasound for a duration of 4 h (up to 35% and 25% reduction of the hydration process time for corn kernels and mung beans, respectively); Ghafoor *et al.* [6] reported up to 33% increase in the water content after treating navy beans to a 750 W, 47 kHz ultrasound for a duration of approximately 4 h; Yang *et al.* [7] reported up to 7% increase in water content after treating soyabean to a 300 W, 40 kHz ultrasound for a duration of 30 min (the water content was measured after 5 days); Yaldagard *et al.* [2] reported up to 126% increase in water content after treating barley seeds to a 460 W, 20 kHz, pulsed ultrasound for a duration of 15 min and subsequently incubated for 36 h. In addition, subjecting the seeds to high-power low-frequency ultrasound can also destroy any microbes present on the seeds because the acoustic cavitation generated from the powerful ultrasound can damage the cell wall and lead to inactivation of microbes [10]. Despite the advantages, the use of high-power low-frequency ultrasounds in consumer products can pose a significant safety risk.

Interestingly, using a higher ultrasound frequency (1 MHz) and lower power (15 W) [3], a similar trend in the increased rate of water absorption in malt radish seeds was reported; up to 20% increase in water content after 24 h. At the maximum power (15 W), the acoustic pressure amplitude was estimated to be approximately 1.3 × 10^4^ Pa, which may be sufficient to induce acoustic cavitation (albeit a weaker one) since tap water was used; the threshold for acoustic cavitation using tap water can be as low as 10^5^ Pa [11]. In this study, we report the enhanced uptake of water in chickpeas and mung beans after treating the seeds to low-power (of the order of 10^0^ W) and high-frequency (on the order of 10^7^ Hz) ultrasound generated using a surface acoustic wave (SAW) device. In this work, the treatment does not induce acoustic cavitation. Based on the power and the frequency of the SAW, although the acoustic pressure amplitude should be of the order of 10^6^ Pa [12], as filtered water is used, the threshold for acoustic cavitation is as high as 10^7^ Pa [11]. Additionally, unlike the high-power low-frequency ultrasound technique in which seeds are fully soaked in water, in our approach, the seeds are only partially in contact with water, thus reducing the amount of water needed in the process.

SAW devices have been used extensively for microfluidic operations and applications such as droplet transport and manipulation [13–16], fluid mixing [17,18], particle concentration [19–21], particle trapping and manipulation [22–25], liquid jetting [26] and atomization [27–30], cooling [31], heating [32], drug delivery [33,34] and nanofiltration [35]. When SAWs travel on the piezoelectric substrate, the surface displacement amplitude of the substrate is in the order of 10^−9^ m, and the amplitude decays exponentially with increasing distance from the substrate surface. When a droplet is placed on the piezoelectric substrate surface in the way of the SAW propagation, due to the difference in the speed of sound in the piezoelectric substrate *c*_SAW_ and the liquid *c*_f_, the acoustic energy is transmitted into the liquid along the Rayleigh angle [36,37], *θ*_R_ ≈ sin^−1^ (*c*_f_/*c*_SAW_). At lower excitation powers (less than 1 W), weak acoustic streaming inside the droplet can generate internal recirculation, which is useful for fluids mixing and particles/cells concentration; whereas at higher excitation powers (more than 1 W), strong acoustic streaming, and unstable liquid–air interface can generate liquid jets and fine droplets, known as atomization. In this study, our focus is on using lower excitation power surface acoustic waves to increase the rate of water absorption in chickpeas and mung beans. Additionally, due to the efficient transmission of acoustic energy from the piezoelectric substrate into the liquid, it is possible to drive the SAW devices using a portable electronic driver circuit. This is a significant advantage (for home appliances) over the high-power (10^2^ W) low-frequency (10^4^ Hz) ultrasound technique which requires heavy equipment that cannot be easily miniaturized.

## 2. Experimental set-up

**Figure 1.**
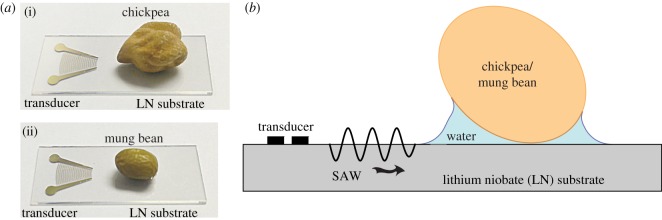
(*a*) Images showing a (i) chickpea and (ii) mung bean on the SAW device. (*b*) Schematic depiction of the side view of the experimental set-up used for enhancing water uptake for seeds. The set-up consists of a lithium niobate (LN) substrate patterned with a focusing-elliptical single-phase unidirectional transducer (FE-SPUDT), to which an input oscillating electrical signal at resonance was applied to generate the SAW. The seeds were partially in contact with water with hilum facing the LN substrate. The sketch is not to scale.

The experimental investigation was split into two parts: (1) effect of SAW on the water absorption for chickpeas and mung beans and (2) effect of SAW on the DNA (deoxyribonucleic acid) of chickpeas and mung beans. Figure 1*a*(i)(ii) shows the brief set-up of the experimental study of the effect of SAW on the rate of water absorption for chickpeas and mung beans, respectively. The SAW devices consist of a 128°-rotated *Y*-cut *X*-propagating single-crystal lithium niobate (LN) piezoelectric substrate (Roditi Ltd, London, UK) on which a focusing-elliptical single-phase unidirectional transducer (FE-SPUDT) was patterned using standard sputter deposition and photolithographic processes. For the FE-SPUDT, the eccentricity is 0.83. A primary function generator (WF1966, NF Corp., Yokohama, Japan) was used to generate a sinusoidal electric signal, which was subsequently amplified using a high-frequency amplifier (25A250A, Amplifier Research Inc., Souderton, PA, USA) and applied to the FE-SPUDT to generate a uniform SAW. The input sinusoidal signal was set at a frequency *f*_SAW_ = 30.5 MHz to match the resonant frequency of the transducer, which is determined by the width and spacing of the transducer fingers; the SAW velocity on LN substrate is approximately *c*_SAW_ ≈ 3990 m s^−1^, thus the SAW wavelength is λ_SAW_ ≈ 131 μm. Prior to the experiment, the initial weight of the dry seeds (chickpea/mung bean) was measured. In each experiment, one chickpea or mung bean was placed at a distance of approximately 5 mm (approx. 38λ_SAW_) from the transducer. Thirty microlitres of water was dispensed at regions between the seed and the LN substrate, as illustrated in figure 1*b*; water was continuously dispensed to replenish the absorbed/evaporated water. Unlike the high-power low-frequency ultrasound, in our experiments with SAW, the seeds were partially in contact with the water; the seed was placed on a pool of water on the LN substrate with the hilum facing the substrate. As noted in the previous study conducted by Kikuchi *et al.* [38], water absorption in seeds was mostly through a hilum. After 60 min, the seed was dried using cleanroom grade paper and the weight of the seed was measured. The change in weight of the seeds was estimated as Δ*W* = (*W*_*c*_ − *W*_*i*_)/*W*_*i*_, where *W*_*i*_ was the weight of the dry seed, and *W*_*c*_ was the weight of seeds after contact with water (with or without SAW). The applied voltage was measured using a voltage probe (TPP 0201, Tektronix, Beaverton, OR, USA) and the electric current using an AC current probe (P6022, Tektronix, USA); both probes were connected to an oscilloscope (TDS 2012C, Tektronix, Beaverton, OR, USA). The electric powers were then calculated as *P*_e_ = *V*_rms_*I*_rms_, where *V*_rms_ was the measured root-mean-square (rms) voltage and *I*_rms_ was the measured rms current. Five different powers—0.16, 0.29, 0.43, 0.62 and 0.86 W—were used in the experiment. The experiment was repeated five times at each power. For each set of data, Student’s *t* probability distribution was employed to calculate the mean and the standard deviation (based on 95% confidence interval), and the two-sample *t*-test was used for comparing two sets of data, using the software MATLAB (MathWorks, USA). By increasing excitation power, some of the mechanical energy is converted into thermal energy, which can affect the water uptake. Hence, the temperature of the water was measured using a T-type thermocouple and the readings were recorded using a data logger (GL820, graphtec, California).

After the completion of the rate of water absorption experiments, we then proceeded to analyse the effect of SAWs on the DNA of the chickpeas and mung beans. The untreated and SAW-treated chickpeas and mung beans were left to germinate for 2 days on a moist pad at room temperature after which the germinated seeds and shoot (if any) were finely ground using a pestle and mortar. The dry chickpeas and mung beans control were ground using a domestic grade grinder. The genomic DNA of the chickpeas and mung beans was extracted using the QIAGEN DNeasy Plant Mini Kit (Hilden, Germany) as per instructions. The concentration of the extracted genomic DNA was measured using a NanoQ (Daejeon, South Korea). A 0.8 wt% agarose gel (Sigma-Aldrich, St. Louis, Missouri, USA) was prepared by adding 50 ml of 0.5X TBE buffer (45 mM Trisborate, 1 mM EDTA, pH 8.4) (Sigma-Aldrich) to 0.4 g of agarose and heating until the agarose fully dissolved. Next, 5 μl of SYBRSafe DNA gel stain (Carlsbad, California, USA) was added and the gel was cast on a casting tray fitted with a well comb. For each sample, 15 ng of genomic DNA and 0.8 μl of 10X gel loading solution was made up to a final volume of 8 μl with 0.5X TBE buffer which was subsequently added to each well. Fifteen nanogram of DNA ladder was added as a reference for each gel. Each sample was carried out in duplicate. The agarose gel was run at 80 mV for 1 h, and visualized using a Biorad Gel-Doc XR+ system (Hercules, California, USA).

## 3. Results and discussion

### 3.1. Water absorption: single seed on LN substrate

**Figure 2.**
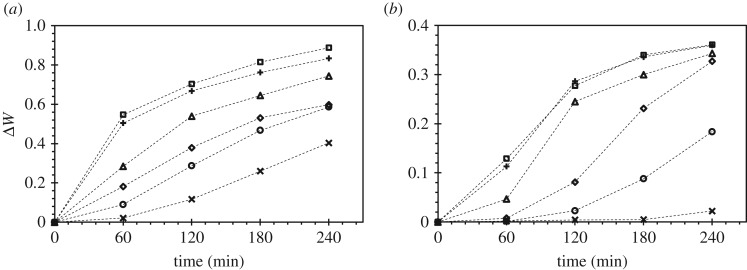
The measured increase in weight over 240 min for (*a*) chickpeas and (*b*) mung beans at different power—(crosses) without SAW (*P*_*e*_ = 0), (circles) *P*_*e*_ = 0.16 W, (diamonds) *P*_*e*_ = 0.29 W, (triangles) *P*_*e*_ = 0.43 W, (+) *P*_*e*_ = 0.62 W and (squares) *P*_*e*_ = 0.86 W—to the transducer.

The water content of chickpeas treated with *P*_*e*_ = 0.86 W SAW, after 60, 120, 180 and 240 min, is up to 2600%, 500%, 210% and 120%, respectively, higher when compared with the seeds without treatment, as shown in figure 2. The results are very interesting since as discussed earlier, previous studies using low-frequency (10^4^ Hz) and high-power (10^2^ W) ultrasound show marginal increases in the water content after 60 min treatment (less than 5%). As reported in the previous study, the enhanced water absorption can be attributed to two main mechanisms [9]: firstly, the formation of micro-cavities due to acoustic cavitation, and secondly, the increase in diffusion rate, which is due to the flow induced by the acoustic wave in the liquid as well as the vibration of the seeds. However, at the low-power (10^0^ W) high-frequency (10^7^ Hz) acoustic waves—the absence of acoustic cavitation—the enhancement in water absorption is possibly attributed to the increase in diffusion rate. An earlier study conducted by Li *et al.* [39] showed that SAW is able to increase the diffusion rate into a porous medium (scaffolds) due to acoustic streaming, i.e. the liquid flow driven by the SAW. Additionally, the seeds are in contact with the water, which enables efficient transmission of the acoustic wave from the water to the seed, giving rise to the propagation of acoustic wave on the seed, which further increases the diffusion rate into the seed.

**Figure 3.**
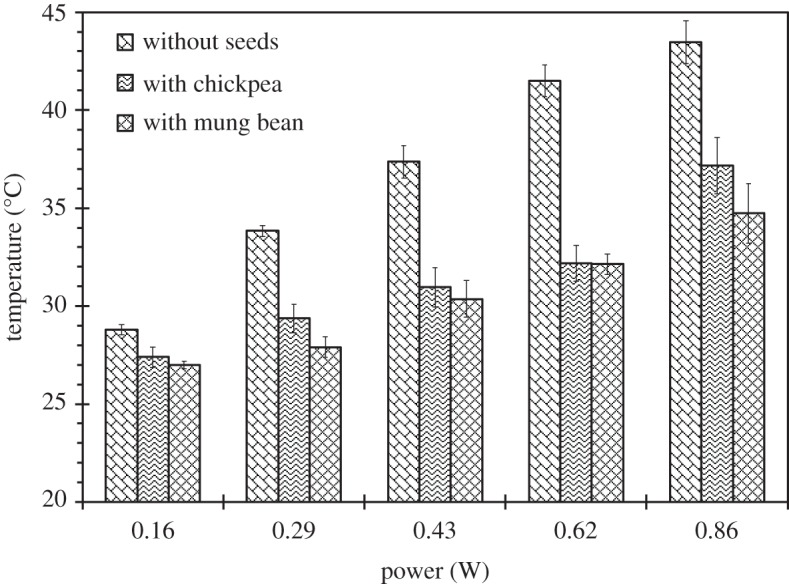
The measured temperature of the liquid, with and without seeds, at different power input to the transducer. Without SAW (*P*_*e*_ = 0), water temperature was approximately 25°C. The error bars indicate the standard deviation of the data taken across triplicate measurements.

While the effect becomes less significant at lower SAW powers, there is still a significant increase in water content for seeds treated with SAW as compared to the seeds without treatment; for *P*_*e*_ = 0.16 W SAW, after 60, 120, 180 and 240 min, the water content of the treated seeds is 350%, 140%, 80% and 45%, respectively, higher when compared with the seeds without treatment. These results show the SAW treatment is particularly effective in the increase of water absorption rate for chickpeas, particularly for a short duration. For example, after 60 min treatment, the difference in the water content between untreated and treated seeds is significant. Nonetheless, from table 1, it can be seen that for the increment/decrement of a power of less than approximately 0.25 W, the difference in the water content is statistically insignificant. Increasing SAW power can increase the temperature of the liquid, which is the consequence of viscous dissipation, i.e. the conversion of mechanical energy into thermal energy [21,40]. Without seeds, water temperature increases at approximately *T* ≈ 22 *P*_*e*_ (°C), whereas with chickpea, water temperature increases at approximately *T* ≈ 13 *P*_*e*_ (°C), as shown in figure 3. The presence of the chickpea reduces the temperature and this can be attributed to the absorption of energy by the chickpea. Without SAW, water temperature stayed constant at approximately 25°C. From the previous study conducted by Shafaei *et al.* [41], the difference in water content between chickpeas soaked in 25°C and 45°C in the absence of any ultrasound, after 4 h, is approximately 10%, suggesting that higher water temperatures enhance diffusion rates. Thus, an increase of approximately 20°C leads to a 10% increase in water content; by comparing that with SAW (*P*_*e*_ = 0.86 W), an increase of approximately 12°C leads to a 120% increase in water content. Hence, the increase in water content is not dominated by the temperature effect but by the acoustic waves.

**Table 1.** Probability values from Student’s *t*-test used to assess the significance of varying the SAW power on the water uptake for chickpea at time (*a*) *T* = 60 min and (*b*) 240 min. *p* < 0.05 denotes statistical significance.

	no SAW	0.16 W	0.29 W	0.43 W	0.62 W
(*a*) after 60 min					
0.16 W	0.108	—			
0.29 W	0.028	0.218	—		
0.43 W	0.011	0.026	0.245	—	
0.62 W	0.007	0.007	0.011	0.057	—
0.86 W	0.002	0.000	0.001	0.013	0.646
(*b*) after 240 min					
0.16 W	0.064	—			
0.29 W	0.009	0.870	—		
0.43 W	0.002	0.111	0.079	—	
0.62 W	0.000	0.010	0.000	0.224	—
0.86 W	0.000	0.004	0.003	0.093	0.284

For mung beans, on the other hand, the increase in water content for the untreated and treated seeds at low power (*P*_*e*_ = 0.16 W) and short duration is not significant. After 60, 120, 180 and 240 min, the water content for treated seeds is up to 0%, 470%, 1660% and 800%, respectively, higher when compared with the untreated seeds. However, a dramatic increase in water content can be seen at high power (*P*_*e*_ = 0.86 W); after 60, 130, 180 and 240 min, the water content for treated seeds is up to 6350%, 6850%, 6700% and 1530%, respectively, higher when compared with the untreated seeds. Thus, unlike chickpeas, where the highest percentage of difference in water content between treated and untreated seeds is at the lowest power *P*_*e*_ = 0.16 W, for mung bean, the highest percentage of difference in water content between treated and untreated seeds is at *P*_*e*_ = 0.29 W. Therefore, higher SAW power is needed to enhance the water absorption for mung bean. Additionally, for an increase or decrease in the power of less than approximately 0.5 W, the difference in water content is statistically insignificant, as shown in table 2. Almost twice as much power is needed for the mung bean to achieve a significant increase in water content when compared with chickpea. As noted earlier, higher SAW power can lead to higher water temperature due to viscous dissipations; with mung bean, it can be seen that water temperature increases at approximately *T* ≈ 11 *P*_*e*_ (°C), a lower rate of increase when compared with chickpea, suggesting that the mung bean absorbs more energy than the chickpea. From the previous study conducted by Liu *et al.* [42], mung beans soaked in water without ultrasound at 25°C and 35° (an increase of 10°C), after 4 h, showed an 200% increase in water content, suggesting that higher water temperatures enhance the diffusion rate. With SAW (*P*_*e*_ = 0.86 W), an increase of approximately 10°C leads to a substantial 1530% increase in water content. Therefore, similar to chickpea, the results suggest that the increase in water content is not due to the temperature effects only, but due to the surface acoustic waves.

**Table 2.** Probability values from Student’s *t*-test used to assess the significance of varying the SAW power on the water uptake for mung bean at time (*a*) *T* = 60 min and (*b*) 240 min. *p* < 0.05 denotes statistical significance.

	no SAW	0.16 W	0.29 W	0.43 W	0.62 W
(*a*) after 60 min					
0.16 W	0.819	—			
0.29 W	0.194	0.181	—		
0.43 W	0.045	0.045	0.065	—	
0.62 W	0.045	0.045	0.052	0.154	—
0.86 W	0.038	0.037	0.043	0.113	0.754
(*b*) after 240 min					
0.16 W	0.034	—			
0.29 W	0.001	0.038	—		
0.43 W	0.000	0.033	0.540	—	
0.62 W	0.000	0.0026	0.286	0.135	—
0.86 W	0.000	0.0019	0.308	0.444	0.955

**Figure 4.**
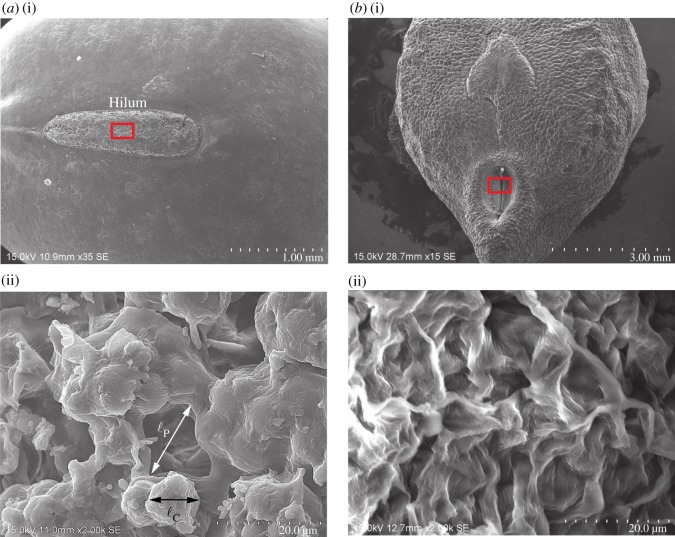
Scanning electron microscope (SEM) images showing: (*a*) a dry mung bean under (i) 35× magnification and (ii) 2000× magnification, and, (*b*) a dry chickpea under (i) 15× magnification and (ii) 2000× magnification. Note that the high magnification images in (ii) were captured at an area highlighted by a rectangular box in (i).

In the presence of SAW, the enhancement in water absorption is significantly higher for mung bean when compared with chickpea. For dry beans, generally, the Poisson ratio is approximately between 0.3 and 0.4, whereas the Young modulus is between 100 and 300 MPa [43]. By assuming a Poisson ratio of *ν* = 0.35, a Young modulus of *E* = 200 MPa and density of *ρ*_B_ ≈ 800 kg m^−3^, the estimated speed of sound in the bean is approximately *c*_B_ ≈ 500 m s^−1^. Note that $c_{B}=\sqrt{K_{B}/\rho_{B}}$, where the bulk modulus *K*_B_ = *E*/[3(1 − 2*ν*)]. Therefore, the specific acoustic impedance for the bean is approximately *r*_B_ = *ρ*_B_*c*_B_ ≈ 0.42 × 10^6^ rayls, whereas the specific acoustic impedance for water is *r*_f_ = *ρ*_f_*c*_f_ ≈ 1.48 × 10^6^ rayls. The percentage of acoustic power transmitted from the water to the bean is approximately $T_{p}=4r_{B}r_{f}/{\left(r_{B}+r_{f}\right)}^{2}\approx 70\text{\%}$. Based on the estimated speed of sound in the bean *c*_B_, and the excitation frequency of *f*_SAW_ = 30 MHz, the acoustic wavelength in the bean is approximately λ_B_ ≈ 17 μm. Note that the acoustic wavelength in the water is λ_f_ ≈ 49 μm. Figure 4*a*(i)(ii) shows images captured using a field-emission scanning-electron-microscope (Hitachi SU8010) for a dry mung bean under 35× and 2000× magnification, respectively. Under 2000× magnification at the hilum of the bean, it can be seen that the size of the cell structure at hilum is larger than ℓ_c_ > 60 μm, whereas the size of the pores is larger than ℓ_p_ > 100 μm. The cell structure size is larger than the acoustic wavelength in the bean (ℓ_c_ > λ_B_), indicating the increase in water absorption is not simply due to the cell structure experiencing expansion and contraction, which is the case when excited by kHz acoustic wave (ℓ_c_ < λ_B_); for MHz excitations (ℓ_c_ > λ_B_), the acoustic wave should be able to propagate on the cell structures, facilitating the transport of water. Additionally, the pore size is approximately the same as the acoustic wavelength in the water (ℓ_p_ > λ_f_), enabling an effective transmission of acoustic wave inside the pores and subsequently the enhancement of water transport within the pores. On the other hand, chickpea has a mesh-like structure (figure 4*b*(ii)), which allows the propagation of the acoustic wave on the structure. However, the pore size is smaller than the acoustic wavelength in the water, which leads to reduction of transmission of acoustic wave inside the pores.

### 3.2. Water absorption: three seeds on LN substrate

**Figure 5.**
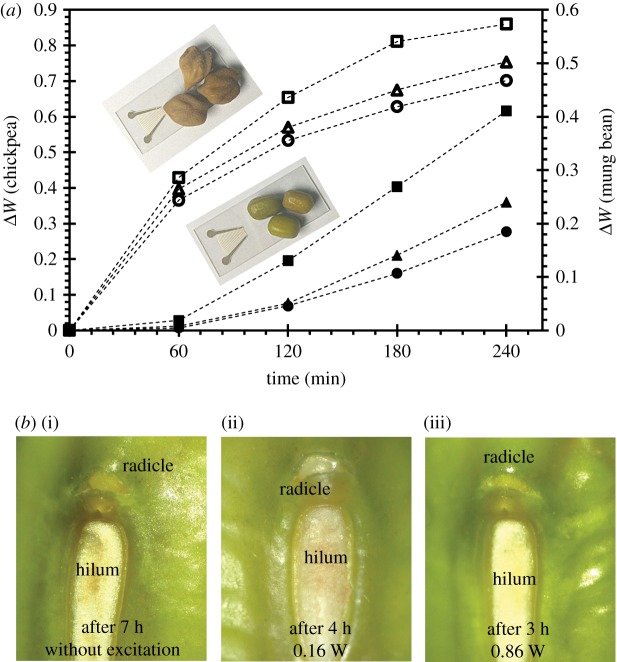
(*a*) The measured increase in weight over 240 min for chickpeas (open circles, open triangles and open squares) and mung beans (filled circles, filled triangles and filled squares) at different power—(open circles and filled circles) *P*_*e*_ = 0.16 W, (open triangles and filled triangles) *P*_*e*_ = 0.43 W, and (open squares and filled squares) *P*_*e*_ = 0.86 W—to the transducer. (*b*) Images showing the comparison of the germinated mung bean between the treated and untreated beans; for (i) untreated bean, radicle emerged after 7 h, for (ii) treated with *P*_*e*_ = 0.16 W SAW, the radicle emerged after 4 h and for (iii) treated with *P*_*e*_ = 0.86 W SAW, the radicle emerged after 3 h.

To demonstrate the feasibility of the technique in water absorption enhancement for multiple seeds in each treatment, here, three seeds were used in each experiment. The experimental set-up and procedures were identical to the single seed as presented in the previous section. Figure 5*a* shows that at the same power to the transducer *P*_*e*_, the water absorption Δ*W* for both chickpeas and mung beans is almost identical to that for the single seed experiment. Therefore, the process remained effective with more seeds on the LN substrate. It is also interesting to note that for mung beans, at high power (*P*_*e*_ = 0.86 W), experiments with a single bean often resulted in no germination, whereas in experiments with three beans, all the beans could still germinate after 4 h exposure. This can be attributed to the lower liquid temperature for the case with three seeds, i.e. at *P*_*e*_ = 0.86 W, the liquid temperature is approximately 3°C–5°C lower for the three beans on LN substrate as compared with single bean on LN substrate. Figure 5*b* shows the increase in water absorption also leads to a faster growth of radicle for the experiments with three beans; higher power SAW *P*_*e*_ = 0.86 W can lead to a reduction of more than 4 h when compared with that without SAW.

### 3.3. Genomic DNA: single seed on LN substrate

As reported by Miano *et al.* [8], seed hydration can be divided into three stages: (1) imbibition phase which is characterized by rapid absorption of water, (2) molecular synthesis where water absorption is minimal and (3) growing of radicle where water absorption is increased. The results shown in figure 2 are related to stage one. Next, the seeds were left for 2 days in the Petri dishes to germinate, i.e. to continue to stage three. Note that the seeds used in this genomic DNA study were from the single seed on LN substrate.

**Figure 6.**
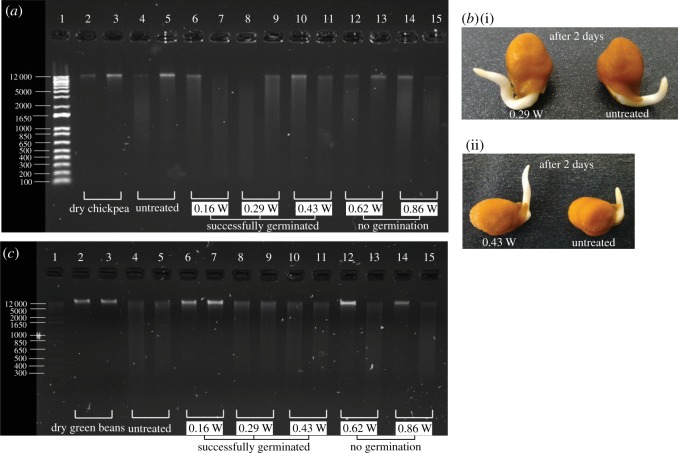
Gel electrophoresis of the genomic DNA of (*a*) chickpea and (*c*) mung bean. (*b*) Images showing the comparison of the germinated chickpeas between the treated and untreated seeds for (i) *P*_*e*_ = 0.29 W SAW and (ii) *P*_*e*_ = 0.43 W SAW after 2 days; radicle is slightly longer for chickpea treated with lower power SAW *P*_*e*_ ≤ 0.43 W. Nonetheless, more samples are needed to quantify the percentage of increase in the radicle length. Note that the samples were from the single seed on LN substrate setup.

The genomic DNA of dry chickpea seeds appeared as a single distinct band on the gel electrophoresis (Lanes 2 and 3, figure 6*a*) compared to germinated untreated seeds which appeared as a smear (Lanes 4 and 5, figure 6*a*). Smearing is indicative of DNA degradation which occurs naturally with successful seed germination [44], and is unlikely to be due to RNA (ribonucleic acid) contamination as any RNA is removed during the DNA purification step in the genomic DNA extraction procedure [45]. Exposure to surface acoustic waves at low powers (0.16 W, 0.29 W and 0.43 W) did not affect the ability of the seeds to germinate. However, the visible failure of the seed to germinate (no radicles), yet smearing of the bands at higher powers at 0.62 W and 0.86 W (bands 12–15, figure 6*a*), indicates possible DNA damage or an incomplete germination process. It is also interesting to note that based on visual inspection, with similar initial weight (difference within ±1 mg), seeds treated with 0.29 W and 0.43 W SAW germinated slightly faster when compared with that without SAW, as shown in figure 6*b*(i) and *b*(ii), respectively. We note here that the slight variation in the duplicate ladders is attributed to differences in the different seeds. For example, in figure 6*a*, Lanes 4 and 5 for the two different untreated chickpeas which germinated, although there is some variation between the ladders, there is distinct smearing of the bands compared to the un-germinated, dry chickpea where no smearing is observed.

Similar results were obtained for the dry mung bean seeds subjected to surface acoustic waves. Distinct bands with minimal smearing were observed for dry chickpeas (Lanes 2 and 3, figure 6*b*) which intensified upon germination (Lanes 4 and 5, figure 6*b*). Exposure of the seeds to low powers of surface acoustic waves between 0.16 W and 0.43 W did not affect the ability of the seeds to germinate as confirmed by the smearing of the DNA band that germination was complete. At higher powers (0.62 W and 0.86 W), however, the seeds failed to germinate (no radicles) and although smearing was observed, this is likely due to genomic DNA damage caused by the heat generated through prolonged exposure to the surface acoustic waves.

## 4. Conclusion

We have demonstrated the increase in the rate of water absorption for chickpeas and mung bean using SAW. Unlike conventional high-power (10^2^ W) low-frequency (10^4^ Hz) ultrasound which often results in acoustic cavitation, using low-power (10^0^ W) and high-frequency (10^7^ Hz) SAW avoids acoustic cavitation. Also, SAW reduces the amount of water required in the process through partial contact with water as compared to the conventional technique where seeds are fully soaked in a water bath. Using high-power SAW (*P*_*e*_ = 0.86 W), after 60 min, the water content of the treated chickpeas is up to 2600% higher than the untreated chickpeas. However, at low-power SAW (*P*_*e*_ = 0.16 W), the increase in water content is reduced to 350% between the treated and untreated chickpeas. This is a significant increase when compared with the 3% difference in water content between the untreated and treated chickpeas obtained using high-power low-frequency ultrasound. Owing to the absence of acoustic cavitation, the increase in the water absorption rate can be attributed to the increase in diffusion rate, which is due to the SAW generated acoustic streaming, and also the propagation of acoustic wave on the seeds. Additionally, we found that the effect of temperature is insignificant, i.e. the increase in the water absorption rate is mainly attributed to the SAW. A minimal increase of 0.25 W is required to obtain a statistically significant increase in water content. For mung bean treated with SAW, after 120 min, the water content of the treated beans is up to 6350% (for *P*_*e*_ = 0.86 W) and 470% (for *P*_*e*_ = 0.16 W) higher than the untreated beans. A similar water absorption rate can also be achieved with three seeds on the LN substrate. For the single seed on LN substrate, for treated seeds with lower powers SAW (*P*_*e*_ ≤ 0.43 W), after 2 days, the seeds can be germinated successfully, whereas for seeds treated with higher powers SAW (*P*_*e*_ ≥ 0.62 W), after 2 days, the seeds failed to germinate, i.e. no radicles can be seen in these seeds. This may be attributed to the higher temperatures of water when the SAW device is excited by higher powers; all seeds are successfully germinated for the three seeds on LN substrate experiments as the water temperature is approximately 3°C–5°C lower when compared with that for the single seed on LN experiments. Finally, our preliminary results also indicate that the treated seeds can germinate faster when compared with untreated seeds. Therefore, our future study will focus on the quantification of the germination rate. In addition, we will also evaluate the optimum configuration that allows more seeds to be treated with minimum power.

## Data accessibility

This article has no additional data.
